# Proton Pump Inhibitors Versus H2 Blockers and the Risk of Incident Dementia in Adults Younger Than 65

**DOI:** 10.7759/cureus.109101

**Published:** 2026-05-18

**Authors:** Nicholas Azinge, Rawan Elkomi, Mekdem Bisrat, Syed Fahad Gillani, Mrinalini Deverapalli, Elizabeth Beyene, Miriam B Michael, Anand Deonarine

**Affiliations:** 1 Internal Medicine, Howard University Hospital, Washington, DC, USA; 2 Internal Medicine, King Edward Medical University, Lahore, PAK; 3 Internal Medicine, Howard University, Washington, DC, USA; 4 Internal Medicine, University of Maryland, Baltimore, USA

**Keywords:** dementia, gastroesophageal reflux disease, h2 receptor antagonists, pharmacoepidemiology, proton pump inhibitors

## Abstract

Background: Proton pump inhibitors (PPIs) and histamine-2 receptor antagonists (H2 blockers) are routinely prescribed for the long-term management of chronic acid-related gastrointestinal conditions. Although prior research has explored a potential link between PPI exposure and cognitive decline, findings across studies have been inconsistent, and most investigations have concentrated on elderly populations.

Objective: This study aimed to evaluate whether PPI therapy, relative to H2 blocker therapy, was associated with differential incident dementia risk among adults below the age of 65 who carry diagnoses warranting sustained acid suppression.

Methods: A retrospective cohort analysis was performed using data from the TriNetX Global Collaborative Network. Eligible participants were adults aged 18-65 with a confirmed diagnosis of gastroesophageal reflux disease with esophagitis (ICD-10-CM K21.0) or Barrett's esophagus (ICD-10-CM K22.7), and documented treatment with either a PPI (ATC code A02BC) or an H2 blocker (ATC code A02BA). All individuals with pre-existing dementia were excluded. Cohorts were balanced via 1:1 propensity score matching on age, sex, and baseline psychiatric and neurodevelopmental diagnoses. The main outcome, incident dementia, was captured through ICD-10 diagnostic codes encompassing Alzheimer's disease (G30), dementia secondary to other diseases (F02), unspecified dementia (F03), and other degenerative nervous system disorders (G31). Analyses included risk ratios, odds ratios, Kaplan-Meier survival curves, and Cox proportional hazards regression.

Results: Following propensity score matching, each treatment group comprised 197,187 patients. New-onset dementia was recorded in 368 individuals (0.19%) assigned to the PPI group and 607 individuals (0.31%) in the H2 blocker group. Relative to H2 blocker use, PPI therapy was linked to a meaningfully lower likelihood of developing dementia (risk ratio 0.61, 95% CI 0.53-0.69; odds ratio 0.61, 95% CI 0.53-0.69). Kaplan-Meier analyses corroborated these findings, with a significantly reduced dementia hazard observed among PPI-treated patients (hazard ratio 0.63, 95% CI 0.55-0.72; log-rank p < 0.001).

Conclusion: In this propensity-matched sample of adults younger than 65 years with chronic acid-related diagnoses, PPI therapy did not elevate the risk of incident dementia relative to H2 blockers and was, in fact, associated with a statistically lower observed risk. These results should reassure clinicians and patients regarding the cognitive safety profile of PPIs when sustained acid suppression is medically necessary.

## Introduction

Proton pump inhibitors (PPIs) and histamine-2 receptor antagonists (H2 blockers) collectively constitute two of the most widely dispensed drug classes globally, serving as cornerstones of treatment for acid-related gastrointestinal disorders such as gastroesophageal reflux disease and peptic ulcer disease [1]. PPIs in particular are frequently continued for extended durations, including in younger working-age adults. Because of their pervasive and often open-ended use, questions about whether long-term PPI exposure might affect neurological health have attracted growing clinical and research attention.

Over the past decade, a series of observational studies raised the possibility that PPIs might promote cognitive decline, particularly in elderly patients [2]. Mechanistic hypotheses have included interference with vitamin B₁₂ absorption, disruption of amyloid peptide processing, endothelial injury, and promotion of neuroinflammatory cascades. Despite these theoretical concerns, the empirical record has been inconsistent; multiple well-powered studies and systematic reviews have yielded null findings or found that apparent associations disappear following more thorough confounding adjustment [3]. A persistent challenge in interpreting the prior literature is that most research has enrolled elderly participants, a group marked by elevated baseline dementia risk, complex comorbidity profiles, polypharmacy, and frailty-all of which complicate causal attribution.

Younger adults differ substantively from elderly patients: their background dementia risk is substantially lower, competing mortality risks are less prominent, and the long-term downstream consequences of medication exposures may follow distinct trajectories. Despite these important differences, evidence directly addressing PPI use and dementia onset in adults younger than 65 remains sparse. A further methodological gap in the existing literature is the relative scarcity of studies employing an active comparator, a design strategy that can substantially reduce confounding by indication when two medications share clinical indications but differ pharmacologically [4].

To address these gaps, we undertook a large propensity-matched cohort study using the TriNetX Global Collaborative Network, directly contrasting incident dementia rates among younger adults managed with PPIs versus H2 blockers [5]. By anchoring analyses in real-world electronic health record data spanning multiple health systems and using active-comparator methodology, this study offers a more rigorous evaluation of acid-suppressive therapy and cognitive outcomes in a population that has received limited attention [6]. It is worth noting that PPIs have been associated with several known adverse effects in some studies, including hypomagnesemia, Clostridioides difficile infection risk, skeletal fractures, and vitamin B₁₂ depletion, though the absolute risk increments for most of these outcomes are modest [7].

## Materials and methods

Study design and data source

This investigation was structured as a retrospective cohort study, drawing on de-identified electronic health records from the TriNetX Global Collaborative Network, a federated, multi-institutional research platform that aggregates demographic, diagnostic, procedural, pharmacological, and longitudinal clinical data from healthcare organizations worldwide. All records comply with applicable data privacy frameworks. Statistical analyses were executed within the TriNetX analytics environment.

Cohort selection

The source population consisted of adults between 18 and 65 years of age at their most recent recorded clinical encounter. Inclusion required a documented diagnosis of either gastroesophageal reflux disease with esophagitis (ICD-10-CM K21.0) or Barrett's esophagus (ICD-10-CM K22.7), coded according to the International Classification of Diseases, Tenth Revision, Clinical Modification (ICD-10-CM) [8].

Participants were assigned to one of two exposure groups according to the medication class documented in their records. The PPI cohort encompassed patients with evidence of any PPI prescription, classified under ATC code A02BC. The H2 blocker cohort captured patients with documented H2 blocker use, classified under ATC code A02BA. The index date was set to the earliest recorded exposure event for the relevant drug class. Individuals carrying any dementia diagnosis antedating the index date were systematically excluded. Dementia was operationally defined using ICD-10 codes for Alzheimer's disease (G30), dementia attributable to other classified conditions (F02), unspecified dementia (F03), and other degenerative diseases of the nervous system (G31). To limit the risk of exposure misclassification, qualifying acid-related diagnoses were required to occur in temporal proximity to the medication record. Patients concurrently exposed to both PPI and H2 blocker agents at baseline were not eligible for inclusion.

Matching and covariate adjustment

Baseline confounding was addressed through 1:1 nearest-neighbor propensity score matching within the TriNetX platform. Variables included in the matching model were age at index date, biological sex, and pre-existing mental, behavioral, and neurodevelopmental diagnoses (ICD-10-CM codes F01-F99).

Propensity scores were derived from a logistic regression model. The matching algorithm applied a caliper constraint of 0.1 pooled standard deviations on the logit-transformed propensity score. Post-matching covariate balance was evaluated via standardized mean differences, with values below 0.1 taken as evidence of satisfactory balance. The resulting matched sample comprised 197,187 participants per group.

Follow-up and outcome ascertainment

Each participant was tracked from the index date forward until the first occurrence of the primary outcome, documented death, or administrative censoring at the end of available follow-up in the network, whichever came first. To guard against reverse causation, outcome capture was initiated one day after the index date. Participants with any dementia-related diagnosis recorded prior to the index date were omitted from all outcome analyses. The primary endpoint was the first coded diagnosis of dementia or a related neurodegenerative disorder during follow-up, using the ICD-10 codes listed above. Mortality was identified through EHR-documented death events and served as a censoring mechanism in time-to-event models.

Statistical analysis

Analyses were performed using the TriNetX platform's built-in statistical tools. Absolute risk (proportion of patients reaching the outcome), risk difference, risk ratio, and odds ratio were each calculated with corresponding 95% confidence intervals. Dementia-free survival was estimated via Kaplan-Meier methods, with between-group differences evaluated using the log-rank test. Cox proportional hazards modeling was applied to generate hazard ratios and 95% CIs, with proportionality assumptions verified within the platform. All hypothesis tests were two-tailed, with a significance threshold of p < 0.05.

## Results

After application of inclusion and exclusion criteria, 498,303 patients in the PPI cohort and 199,887 patients in the H2 blocker cohort met eligibility criteria prior to matching. Following 1:1 propensity score matching, 197,187 patients remained in each cohort with balanced baseline characteristics.

The mean age and sex distribution were comparable between cohorts after matching. Standardized mean differences for matched variables were below 0.1, indicating adequate covariate balance (Table 1).

**Table 1:** Baseline Characteristics of the Study Population. PPI: Proton pump inhibitor

Characteristic	PPI Cohort (n = 8, 539, 024)	H2 Blocker Cohort (n = 8, 539, 024)
Age at index, mean ± SD (years)	40.2±14.8	40.1±15.5
Female sex, %(n)	56.9% (4,858,704 )	55.8% (4,764,775)
Male sex, %(n)	43.1% (3,680,319)	44.2% (3,774,248)
Mental, behavioral, and neurodevelopmental disorders, %(n)	34.2% (2,920,346)	34% (2,903,268)

Incident dementia

During follow-up, incident dementia was diagnosed in 368 patients (0.19%) in the PPI cohort and 607 patients (0.31%) in the H2 blocker cohort.

Risk analysis demonstrated a lower risk of dementia among patients treated with PPIs compared with H2 blockers, corresponding to a risk ratio of 0.606 (95% CI 0.532-0.69) and an odds ratio of 0.605 (95% CI 0.532-0.689) (Table 2).

**Table 2:** Risk of Incident Dementia Propensity scored matched analysis results PPI: Proton pump inhibitor

Outcome	PPI Cohort	H2 Blocker Cohort	Risk Difference	Risk Ratio	Odds Ratio	Hazard Ratio (95% CI)	p-Value
Sample size (n)	8,539,024						
Incident dementia	368	607	0.001	0.606(0.532-0.69)	0.605(0.532-0.689)	0.631(0.554-0.718)	<0.001

Kaplan-Meier survival analysis showed a lower cumulative incidence of dementia in the PPI cohort compared with the H2 blocker cohort. Cox proportional hazards modeling demonstrated a hazard ratio of 0.63 (95% CI 0.55-0.72), with log-rank testing indicating statistical significance (p < 0.001). Median dementia-free survival was not reached in either cohort during the available observation period.

## Discussion

In this large, propensity-matched, multicenter analysis, adults under 65 years of age who were treated with PPIs demonstrated a lower documented likelihood of developing dementia relative to those managed with H2 blockers. The direction and magnitude of this association were reproducible across absolute risk metrics, relative risk estimates, and time-to-event methodology, lending robustness to the overall pattern [9].

The present findings contribute meaningfully to ongoing scientific debate over the neurocognitive implications of PPI therapy. Early signals of potential harm emerged from observational studies conducted primarily in elderly cohorts burdened by multiple comorbidities and substantial competing risks [9]. More recent investigations have reported a heterogeneous set of results, with several well-designed analyses documenting no significant association after thorough confounder control [10,11]. A major advantage of the current approach is its focus on a younger patient group in conjunction with an active-comparator design; together, these features address key sources of bias that have undermined the interpretability of older studies, including confounding by age-related frailty and differential patterns of healthcare engagement.

A range of biological pathways has been postulated to link PPI use to cognitive harm, among them impaired cobalamin absorption, altered processing of amyloid precursor proteins, microvascular endothelial injury, and systemic neuroinflammation [12,13]. Each of these mechanisms remains largely theoretical, and most may be more biologically plausible in aged individuals with established vulnerability to neurodegeneration. The absence of any elevated dementia signal among younger PPI users in this analysis raises the possibility that these proposed pathways either do not translate into clinical consequences in this population or that earlier associations in older cohorts reflected inadequately controlled confounding rather than direct pharmacologic toxicity.

The active-comparator framework represents a central strength of this work. Comparing PPI users with non-medicated individuals is inherently susceptible to confounding by indication: patients prescribed PPIs may differ systematically from untreated individuals in ways that independently influence dementia risk [14]. By instead benchmarking PPIs against H2 blockers, medications prescribed for substantially overlapping clinical indications but with distinct mechanisms of action, the analysis minimizes this source of bias and more closely adheres to current standards in pharmacoepidemiology [15]. Restricting eligibility to patients with diagnoses that clinically justify ongoing acid suppression adds a further layer of exposure validity and reduces contamination from incidental or transient medication use.

Drawing on a large, federated, real-world EHR dataset spanning diverse health systems enhances the external generalizability of these findings [16]. When considered alongside existing randomized and observational evidence, the results support the view that dementia risk in younger adults should not be cited as a primary rationale for withholding PPI therapy when it is otherwise appropriate.

Clinical implications

The results of this study offer actionable guidance for clinicians who prescribe acid-suppressive therapy to younger adult patients. Among individuals with comparable baseline profiles and well-established clinical indications for chronic acid management, PPI treatment was not accompanied by elevated dementia incidence and was, by relative comparison, associated with fewer dementia events than H2 blocker treatment.

For practitioners managing patients below age 65 who require sustained acid suppression, these data provide meaningful reassurance: apprehension about early-onset dementia risk should not, in isolation, discourage clinically appropriate PPI prescribing. Therapeutic selection should remain grounded in symptom severity, endoscopic or histologic disease burden, and established gastrointestinal indications. The active-comparator methodology employed here supports a nuanced understanding of comparative medication safety that moves beyond undifferentiated avoidance of a heavily utilized drug class.

Limitations

Several important constraints should be considered when interpreting these findings. The retrospective, administrative-data design introduces the possibility of incomplete documentation, cross-institutional variability in coding practices, and diagnostic misclassification. Dementia ascertainment relied on ICD-10 billing codes rather than structured clinical evaluation, potentially missing subclinical or early-stage cases-an issue that may be particularly pronounced in younger adults, among whom dementia tends to be underdiagnosed and less frequently coded.

Medication exposure was indexed on prescription records, without access to dispensing logs, adherence metrics, cumulative dosing data, or over-the-counter supplement use. Although cohort inclusion was restricted to patients with conditions consistent with sustained acid-suppressive therapy, an approach intended to approximate chronic exposure, precise treatment duration could not be ascertained, leaving some exposure misclassification likely. Propensity score matching, while effective for addressing measured confounding, cannot eliminate bias from unmeasured factors. Variables such as socioeconomic position, educational background, lifestyle characteristics, family history of dementia, and healthcare-seeking behaviors were unavailable in the dataset and may have differentially influenced group assignment and outcomes.

As with all observational research, the study cannot establish causality. While the active-comparator design and restriction to clinically relevant indications strengthen internal validity, the findings should be characterized as associational rather than as evidence of a protective or harmful pharmacologic effect attributable to PPI therapy per se.

## Conclusions

In this propensity score-matched cohort of adults under 65 with established indications for acid-suppressive therapy, PPI use was not associated with elevated dementia risk compared to H2 blockers and, in fact, demonstrated consistently lower dementia incidence across all analytic approaches, strengthening confidence in the observed effect despite low absolute event rates. While prior concerns stemmed largely from elderly, comorbidity-burdened populations, the present study's focus on younger adults with clinically justified exposures reduces heterogeneity and indication-driven bias, and the active-comparator design further enhances inferential validity. Though causal conclusions cannot be drawn from observational data, these findings offer no support for the hypothesis that PPIs accelerate dementia onset and should reassure clinicians that cognitive risk alone is not a well-founded reason to withhold appropriate PPI therapy, with future studies in older populations incorporating dose and duration data needed to complete the long-term neurocognitive safety picture.
